# Supplementary material for: Chromothripsis during telomere crisis is independent of NHEJ, and consistent with a replicative origin
Source: Genome Res. 2019 May;29(5):737–49. doi: 10.1101/gr.240705.118 (PMC6499312; doi:10.1101/gr.240705.118)
Supplement: Supplemental Material [file supp_gr.240705.118_Supplemental_file_1.zip › contigs/annotated_contigs/DB115/contig.2.DB115_length_753_mean_cov_7.37051792829.docx]

**DB115_length_753_mean_cov_7.37051792829**

```
TTACTGCCAAATACTTGGTTCACACAGTTATCCCAGGCCCTCCACACAATAGCTCTGTGCCTTTTCTAAGCTTGACCTTCTTTATCCTA
 >chr14:30312932-30313318 - E=2e-220
AAATAGGTTTATTTTTCTCACTATTAGTATCTTGATGATAAATGAGATTATATTTATACATTTTTTACTACAATACTGGATCCAAGGTT

GACATGCTTAATTGCCTTCAAGTCTGCTCGAATGTCATCTTTTCATTGAAGCCTGTCCTGCAGCTGTGTTTAAGTTGCAAACAACTTTT

ACTATTACTCTTAATCTCCCATACTGTTTTCTCTATAACATTTACCATTTTCTAACATACTTTATAACTCATTTATTTATTATTAGGTC

TATTCATTATGTGTTACTCTGCACCAGAG|T|AGGACTTCAATACACACATTTTGGGGATAAACAATTCCGTTAATAATACCTTCTATT
                              >chr14:30311910-30312278 - E=1e-203
TCTCTGGCTGGCTCTTTCTATAAGTTGCTTGGTTGGGATGATTTGTTTGAGTTTTTGTTTACCATTTCACTTTTGGATACTGTCTTTTT

CATGACCTTTTGCTCCAAAGTTTGAGTTGCCAGCCATTCTTTCTTCCCTGCTATACCCGCTCCCCACATTTAACCCTCTGCCTGGTCCA

TACTCCCAGGAGTCTTTCAGAAAAAGCGGTTCAAAGGAAAGACTATTTTCATGCTTGGTATGCTCATAATCCTGTAGACATATCAGTTT

GGAGGCTAACATGTGTCAACTGATGGACAATTAGCATACAGAA
```
